# Disease modification and Neuroprotection in neurodegenerative disorders

**DOI:** 10.1186/s40035-017-0096-2

**Published:** 2017-09-26

**Authors:** Jeffrey Cummings

**Affiliations:** Cleveland Clinic Lou Ruvo Center for Brain Health, 888 W Bonneville Ave, Las Vegas, NV 89106 USA

**Keywords:** Alzheimer’s disease, Frontotemporal dementia, Progressive supranuclear palsy, Corticobasal degeneration, Amyotropic lateral sclerosis, Multiple system atrophy, Disease modification, Disease modifying therapy

## Abstract

**Background:**

Disease modifying therapies (DMTs) are urgently needed for neurodegenerative diseases (NDD) such as Alzheimer’s disease (AD) and many other disorders characterized by protein aggregation and neurodegeneration. Despite advances in understanding the neurobiology of NDD, there are no approved DMTs.

**Discussion:**

Defining disease-modification is critical to drug-development programs. A DMT is an intervention that produces an enduring change in the trajectory of clinical decline of an NDD by impacting the disease processes leading to nerve cell death. A DMT is neuroprotective, and neuroprotection will result in disease modification. Disease modification can be demonstrated in clinical trials by a drug-placebo difference in clinical outcomes supported by a drug-placebo difference on biomarkers reflective of the fundamental pathophysiology of the NDD. Alternatively, disease modification can be supported by findings on a staggered start or delayed withdrawal clinical trial design. Collecting multiple biomarkers is necessary to support a comprehensive view of disease modification.

**Conclusion:**

Disease modification is established by demonstrating an enduring change in the clinical trajectory of an NDD based on intervention in the fundamental pathophysiology of the disease leading to nerve cell death. Supporting data are collected in clinical trials. Effectively defining a DMT will assist in NDD drug development programs.

## Background

Alzheimer’s disease (AD) and many other neurodegenerative disorders (NDD) are becoming more frequent as the world’s population ages; they constitute an impending public health catastrophe. AD doubles in frequency every 5 years after the age of 65 and affects up to 50% of those over age 85 [1]. Similarly, Parkinson’s disease (PD) and amyotrophic lateral sclerosis (ALS) are age-related conditions and are increasing in frequency with the aging of the population [2, 3]. Progressive supranuclear palsy (PSP), corticobasal degeneration (CBD), frontotemporal dementia (FTD), dementia with Lewy bodies (DLB), Huntington’s disease (HD), multiple system atrophy (MSA), chronic traumatic encephalopathy (CTE), spinocerebellar ataxias and a variety of more rare proteinopathies are NDD. Cumulatively, NDD affect millions of individuals worldwide, costing governments billions of dollars in healthcare expenditures and lost economic productivity [4]. Treatment to ameliorate the social and personal tragedy of NDD is an urgent global priority.

NDD are protein aggregation disorders with neurodegeneration [5]. Neurodegeneration is defined as progressive loss of neurons and their processes with a corresponding progressive impairment in neuronal function [6]. Advances in neuroscience have provided new insights into NDD including their many shared features [7]. Impaired autophagy, protein aggregation, inflammation, oxidative injury, genetic and epigenetic features, mitochondrial impairment, apoptosis, reduced growth factor effects, and loss of synaptic plasticity are involved across NDD. Differing phenotypes of NDD reflect regional central nervous system (CNS) patho-geographies based on the molecular characteristics of the aggregating protein and genetic and environmental influences. PD is associated with substantia nigra and related regional changes; FTD with asymmetric frontal and temporal alterations; HD with striatal impact; and AD with initial medial temporal effects. The progression of NDD is increasingly understood in terms of prion-like transmission of proteins along disease-relevant pathways [8]. Degeneration in NDD occurs in networks of related neurons as identified by functional magnetic resonance imaging (fMRI) studies. These networks also serve as pathways for the propagation of proteins that aggregate in the course of NDD [9, 10].

Despite progress in understanding NDDs, there is a marked translational gap from biology to treatment and no disease-modifying therapy (DMT) has been approved for any NDD [11–15]. Aggressive efforts are required for seeking candidate molecules with possible disease-modification (DM) effects, developing new animal models that more accurately predict human efficacy, and advancing clinical trial methods and conduct [11, 16–18].

Among the clinical trial issues to be addressed in this important enterprise is the definition of DMT. Sponsors cannot plan development program without a clear understanding of how DMTs are defined, and trials cannot be designed and outcome measures chosen without such a definition. Preliminary work on defining DMTs has been accomplished by regulatory agencies that govern drug labeling (U.S. Food and Drug Administration [FDA]; European Medicines Agency [EMA]) and broad consensus building is needed to insure agreement among stakeholders representing trial design, biomarkers, biostatistics, regulation, commercial development, and patients and families. Cummings and Fox [19] recently offered a preliminary definition of DMT for AD. Here, the concept of neurodegeneration, DMT, the relationship of DM to neuroprotection, and the application of the definition to multiple NDDs are described.

### Definition of Neurodegeneration and disease-modifying therapy

Neurodeneration is defined as loss of neurons and their processes (axons, dendrites, synapses) with a corresponding progressive impairment in neuronal function [6]. A DMT is defined as an intervention that produces an enduring change in the clinical progression of an NDD by interfering in the underlying pathophysiological mechanisms of the disease process leading to nerve cell death [19]. Two types of data supporting an intervention as a DMT have been identified: 1) the intervention produces a significant drug-placebo difference on accepted clinical outcome(s) and has a consistent effect on biomarkers considered reflective of the fundamental pathophysiology of an NDD, or 2) the intervention produces a positive outcome on a staggered start or delayed withdrawal clinical trial design consistent with an enduring change in clinical course. In both cases, slowing of disease progression on clinical measures is evident; in the former, biological evidence of DM supports the clinical measures.

### Disease modification and Neuroprotection

**Fig. 1:**
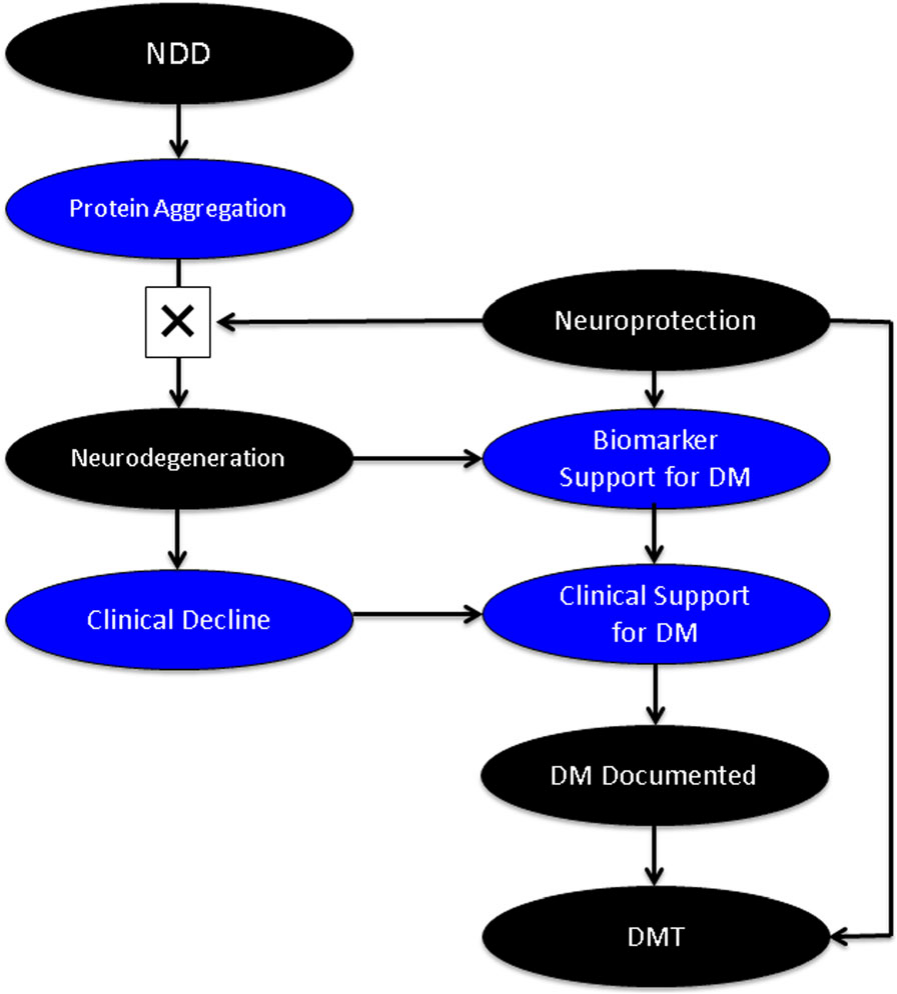
The conceptual framework for disease-modification in neurodegenerative disorders included neuroprotection preventing neurodegeneration with biomarker and clinical support for a disease-modifying therapy (DMT)

The effect of a DMT is to produce an enduring change in the trajectory of clinical decline of the NDD by impacting the disease processes leading to neuronal death [19]. Neuroprotection is defined as an intervention that favorably influences the disease process or underlying pathogenesis to produce lasting benefits for patients [20, 21]. Effective neuroprotection results in disease modification and efficacious neuroprotective therapies are disease-modifying (Fig. 1).

Neuroprotection might be achieved by direct effects on neurons (primary neuroprotection) or by interfering with processes that lead secondarily to cell death such as inflammation. The final common pathway to be achieved for DM is neuroprotection.

### Biomarker support for disease-modification

Most development programs rely on biomarkers to provide support for DM. The biological change associated with DM is neuroprotection and biomarker support for DM depends on demonstration of neuronal preservation. DM and neuronal preservation cannot be observed directly and must be inferred from other types of evidence including biomarkers. To support DM, the biomarker must be indicative of a change in the processes leading to the loss of neurons.

**Fig. 2:**
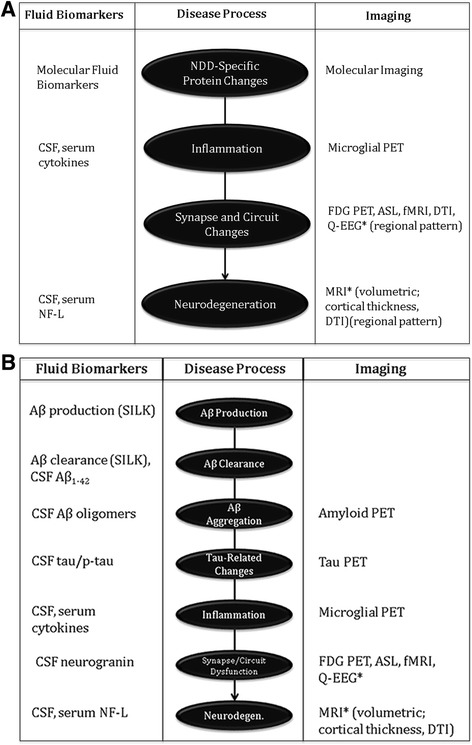
Biomarkers of neurodegenerative disorders (**a**) and of Alzheimer’s disease (**b**). The exact order of the cascade of events from Aß production to cell death is not fully determined (Aß – amyloid ß protein; AD – Alzheimer’s disease; ASL – arterial spin labeling; DTI – diffusion tensor imaging; FDG – fluoroeoxyglucose; fMRI functional magnetic resonance imaging; NF-L – neurofilament light; PET – positron emission tomography; p-tau – phosphorylated tau protein; Q-EEG – quantitative electroencephalography; SILK – stable isotope-labeled kinetics). *The regional pattern of these changes may be specific to the neurodegenerative disease

Evidence regarding which biomarkers reliably reflect processes leading to cell death is evolving (Fig. 2). Cell death is the end product of a cascade of events and which elements of the cascade are critical to measure in support of DM has not been completely ascertained. Given the uncertainties regarding biomarkers as reporters of DM, the FDA has advised collecting multiple biomarkers in the course of trials to support a drug effect on the underlying biology of the NDD. In its guidance on “Alzheimer’s Disease: Developing Drugs for Treatment of Early Stage Disease” [22] the FDA noted that a biomarker effect must reflect a pathophysiological entity that is fundamental to the underlying disease process. They observed that there is currently insufficient evidence on which to base a hierarchical structuring of biomarkers and encouraged trial sponsors to analyze the results of multiple biomarkers independently.

Magnetic resonance imaging (MRI) is a measure of brain volume and shows increasing brain atrophy with progression of AD and other NDD [23–25]. MRI is a candidate biomarker for DM. In AD clinical trials, however, MRI has often shown greater atrophy in active treatment than placebo groups and its ability to function as a biomarker to support DM has not been shown [26, 27]. Diffusion tensor imaging (DTI) assessments are candidate measures for loss of spiny neurons and their projections in HD [28]. Measures of iron and neuromelanin have promise as MRI outcomes assessing neurodegeneration in PD [29], and a combined measure of third ventricle, midbrain, and frontal lobe has been proposed to measure DM effects in clinical trials in PSP [30]. Brain boundary shift intervals and ventricular boundary shift intervals correlate with disease progression in FTD and may perform well in clinical trials to show drug-placebo differences in atrophy with an implied effect on neurodegeneration [24].

In AD, removal of plaque amyloid has been demonstrated with several immunotherapies with no corresponding clinical benefit and reducing fibrillar amyloid demonstrated with amyloid imaging is not by itself support of DM [31, 32]. Removal of plaques was associated with a beneficial impact on cognitive decline in studies of aducanumab, suggesting that fibrillar amyloid removal may correlate with DM in some therapeutic circumstances [33]. Effects on soluable forms of amyloid may be key to establishing DM with anti-amyloid therapies in AD [34].

Tau PET detects neurofibrillary tangles closely associated with cell death and may serve as a marker for neurodegeneration in AD [35, 36]. Some tau-related tracers may be valuable as measures of neurodegeneration in tauopathies such as CTE [37]. High CSF tau levels predict cognitive decline in AD, PD, and Creutzfeldt-Jakob disease [38–40]. These observations demonstrate a relationship between CSF tau and clinical course suggesting that drug-placebo differences in tau elevation may be one means of supporting DM with a biomarker. CSF tau is not abnormal in all NDD; in addition, changes in tau might not be equally supportive of DM across all phases of NDDs.

Emerging biomarkers may assist in supporting DM in DMT trials. Measures of neurofilament light chain protein show the protein to be increased in CSF of patients with AD, FTD, and ALS [41, 42] suggesting that it may be a marker of neurodegeneration relevant to several NDD populations. Drug-placebo differences in elevation of this peptide might offer support for DM. VILIP-1 and neurogranin are additional proteins shown to be abnormal in AD and to predict cognitive decline [43, 44]. These biomarkers may assist in demonstrating DM in AD.

The principal biological means by which DM will be achieved are uncertain and how the intervention will be reflected in serum, CSF, or imaging biomarkers is currently unprooven. Biomarker data of multiple types indicating a consistent, robust effect on fundamental disease pathophysiology in concert with compelling clinical information is most likely to demonstrate that an agent is a DMT. Biomarkers from multiple classes – neurodegeneration, biomarkers specific to the agent’s mechanism of action – are more consistent with DM than measures within a single class of biomarkers [19]. Biomarkers may be specific to a disease or reflective of the general neurodegerative process and present across multiple disease states (Fig. 2). Measures of Aß are specific to AD, alpha-synuclein measures are abnormal in PD and DLB, and measures of Huntington or TDP-43 would be reflective of HD and FTD or ALS, respectively. Changes in brain metabolism (fluorodeoxyglucose) or circuit function (functional MRI) may have regional patterns specific to disease-states [45, 46]. Interventions at any level of the cascade leading to cell death may be neuroprotective and preventive of neurodegenation. Such agents would be DMTS. Biomarkers assist in identifying the presence, type, and magnitude of change induced by therapy.

### Trial designs to support disease-modification

**Fig. 3:**
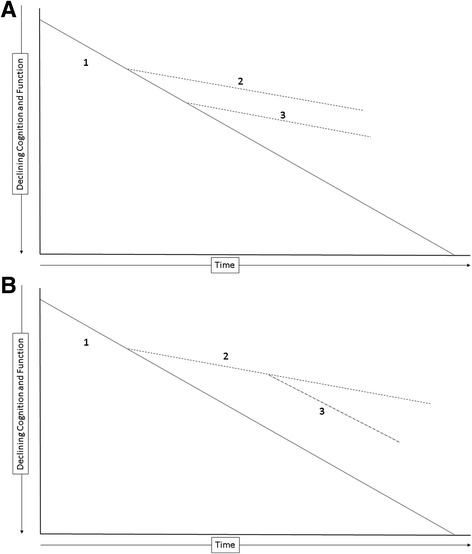
Delayed start design (**a**). The delayed treatment group (3) does not catch up with the early treatment group (2). (1) is the untreated, placebo group. Staggered withdrawal design (**b**). The withdrawn group (3) does not decline to the level of the placebo group (1)

The central feature of a DMT is an intervention that produces an enduring change in the course of NDD such that if the treatment is interrupted the patient does not resume the same level of function as one not treated. The concept of enduring change is embedded in the staggered start and delayed withdrawal clinical trial designs [47–50] (Fig. 3). In the staggered start trial, the participants in the delayed therapy group do not catch up with the initial therapy group if the treatment is a DMT. Catching up would be expected with a symptomatic therapy and not with a DMT since the course of the original group has been permanently altered by the DMT. Similarly, in the delayed withdrawal design, one group is withdrawn from therapy to determine if they assume the level of function of a placebo-treated group. Failure to decline to the untreated level is evidence of DM, whereas returning to the same level as an untreated group suggests that the treatment under study has symptomatic effects without DM [22, 48, 49, 51, 52].

These trials are cumbersome, the duration of periods of observation off drug to draw comparisons with the treatment group, ethical challenges to withdrawing patients from treatment, and statistical issues related to adequate powering of the studies have all contributed to lack of use of these designs to support DM. The ADAGIO study in PD used a staggered start design [53]. A benefit was observed with the 1 mg dose and not the 2 mg dose leading to ambiguity in interpreting the trial in favor of DM by rasagiline [54]. Advances in analytic approaches to two-period designs may improve the utility of these designs and broaden their application [50].

Staggered start analyses have been used at trial termination when patients on placebo for the double-blind portion of the trial are switched to active therapy. This provides an opportunity to see if the placebo-treated patients catch up with those on treatment throughout the trial. This approach was used for analysis of the open label extension data of the Expedition and Expedition II data of solanezumab trials for treatment of AD [55]. The analysis suggested continued benefit, although DM was not confirmed in a subsequent trial [56]. This approach to a staggered start is compromised by the open-label nature of the analysis population (i.e., knowledge by patients and investigators that all patients are on active treatment in the extension period). The observation might help support DM in conjunction with other analyses and biomarker outcomes.

### Disease-modification applies across neurodegenerative disorders

NDD are protein aggregation disorders with neurodegeneration [5]. AD, PD, PSP, CBD, FTD, ALS, DLB, HD, MSA, CTE, spinocerebellar ataxias and a variety of more rare proteinopathies are NDD [57, 58]. In all cases the same principles of DM, neurodegeneration, neuroprotection, and defining a DMT apply. Biomarkers, trial designs, many aspects of the underlying neurobiology, regulatory reviews, and even some interventions apply across NDD [7]. To achieve DM in any of these NDD, neuroprotection must be achieved. The enduring change critical to the concept of DM can be demonstrated by clinical measures plus biomarkers or by DM-type of trial designs. Some biomarkers such as neurofilament light chain protein may reflect later stages of cell death applicable to several NDD [41, 42]. Use of other biomarkers will vary depending on the pathobiology of the specific NDD; disease-specific biomarkers typically reflect events earlier in the cascade of processes leading to cell death [59] (Fig. 2). Biomarkers of target engagement are critical in drug development but do not necessarily imply neuroprotection and may not be supportive of DM.

## Conclusion

DMTs are urgently needed to address the global public health crisis posed by AD and other NDD. NDD are steadily progressive clinically, reflecting an on-going loss of nerve cells and corresponding brain dysfunction. The biological changes are reflected in biomarkers. DM can be shown by a change in clinical course and corresponding alteration in fluid and imaging biomarkers. The enduring change in disease trajectory can also be demonstrated by staggered start or delayed withdrawal clinical trial designs. DMTs are neuroprotective and neuroprotection is the basis for DM. Neuroprotection may be achieved through interruption of upstream NDD-specific processes leading to cell death or through affecting downstream processes reflecting the intracellular final common pathway of neuronal death. Biomarker changes associated with successful DM may reflect the mechanism of action of the agent, level of interruption, and impact on downstream processes. Target engagement biomarkers may not equate to DM. A repertoire of biomarkers is needed to understand the full impact of a DMT. Implementing the definition and collecting evidence in support of DM will facilitate the development of DMTs.
